# Intranasal administration of resveratrol successfully prevents lung cancer in A/J mice

**DOI:** 10.1038/s41598-018-32423-0

**Published:** 2018-09-24

**Authors:** Aymeric Monteillier, Aymone Voisin, Pascal Furrer, Eric Allémann, Muriel Cuendet

**Affiliations:** School of pharmaceutical sciences, University of Geneva, University of Lausanne, Rue Michel-Servet 1, CH-1211 Geneva 4, Switzerland

## Abstract

Lung cancer is the most lethal cancer in the world. About 80% of lung cancer deaths are linked to tobacco use. As a complement to tobacco control, efficient chemoprevention strategies are needed to tackle lung cancer epidemic. Resveratrol is one of the most studied natural products, notably for its cancer chemoprevention properties. However, its low oral bioavailability has often limited the translation of *in vitro* activities to *in vivo* effects. While oral administration of resveratrol effectively inhibited colorectal carcinogenesis, it failed to protect mice from chemically-induced lung carcinogenesis. Therefore, non-invasive parenteral routes must be considered to bring resveratrol to the lungs. In the present study, intranasal administration of a concentrated formulation proved to be a valid method to expose the lungs to a sufficient amount of resveratrol. This formulation was administered three times a week for 25 weeks to A/J mice having 4-[methyl(nitroso)amino]-1-(3-pyridinyl)-1-butanone-induced lung carcinogenesis. Resveratrol-treated mice showed a 27% decrease in tumour multiplicity, with smaller tumours, resulting in 45% decrease in tumour volume/mouse. *In vitro* investigations highlighted apoptosis as a potential mechanism of action. This study presents an effective way to overcome resveratrol low oral bioavailability, encouraging a reevaluation of its use in future clinical trials.

## Introduction

Lung cancer is the worldwide leading cause of cancer death, accounting for 1.69 of the 8.8 million global cancer deaths in 2015. Behind these striking numbers, tobacco smoke stands as the major risk factor and is responsible for about 80% of those deaths^1^, highlighting the preventability of most lung cancers. Despite the general decrease in smoking prevalence due to growing tobacco control strategies^2^, the global number of smokers continues to rise every year and is estimated to reach about 1.1 billion in 2025^3,4^. Although efficient tobacco control strategies, such as the WHO Framework Convention on Tobacco Control, are essential to fight the tobacco smoke epidemic and the associated lung cancer deaths, their implementation takes time and will probably mainly benefit future generations. A complementary approach is therefore needed to prevent those deaths, involving the development of both early cancer diagnosis and chemoprevention strategies. While considerable advances have been made in early diagnosis, mainly through low dose computed tomographic screening, which was able to reduce the mortality from lung cancer by 20%^5^, lung cancer chemoprevention strategies are still under development and none are yet validated for clinical use^6^. Considering that a chemopreventive drug would be administered to persons at risk during a long period for a disease they are not sure to develop, it should have a limited impact on their daily life. This implies a very good toxicity profile with none or minor adverse effects, as well as a convenient pharmaceutical form and administration route. Up to now, most of the 12 FDA approved cancer chemopreventive agents do not totally fulfill these requirements in terms of toxicity, therefore restricting their use to a small part of the population for whom the high cancer risk justifies the drug adverse effects. A good example is the use of tamoxifen, a selective estrogen receptor modulator that, despite its ability to decrease up to 50% the probability of developing breast cancer, is limited to women at high risk due to increased probability of developing endometrial cancer or thrombotic events^7^. Another example is the use of nonsteroidal anti-inflammatory drugs (NSAIDs) for colorectal cancer prevention, which is limited by the gastrointestinal bleeding that they promote. Finding agents with a better toxicology profile could extend their use to a greater number of patients, hence increasing the coverage and the preventive efficacy.

An important target in lung cancer chemoprevention is tobacco-induced inflammation. Among the 12 FDA approved drugs for cancer chemoprevention, three are NSAIDs (aspirin, celecoxib, diclofenac sodium)^6^. Inflammation is widely recognized as a tumour promoter, mainly through an overactivation of the nuclear factor kappa B (NF-κB) pathway. This was observed in both preclinical models and in human patients, and resulted in the expression of genes involved in cell survival, proliferation, mobility and angiogenesis^8–11^. NF-κB was necessary for the development of lung adenocarcinoma in mice, and its inhibition induced tumour regression^12,13^. Among the natural compounds that combine anti-inflammatory properties through NF-κB inhibition and a safe toxicology profile, resveratrol (3,5,4′-trihydroxystilbene, RES) is probably the most extensively studied. This phytoalexin, produced by a great variety of plants and present in numerous food products such as grapes, peanuts and berries, has generated a huge interest since the discovery of its cancer chemopreventive activity 20 years ago^14^. While the amount of *in vitro* data generated since then confirmed its activity in various types of cancers, *in vivo* studies have often been damped down by the poor pharmacokinetic profile of RES^15,16^. When taken orally, RES is indeed highly absorbed through the gastrointestinal tract but quickly and extensively metabolized in the liver and then excreted in the urine. Therefore, only a minor fraction of the absorbed RES will eventually reach the internal organs, making its activity dependent on the administration route^17^. Several studies showed that oral administration of RES effectively inhibited carcinogenesis in the digestive tract, but failed to protect mice from chemically-induced lung carcinogenesis^18–21^. In this context, other administration routes must be considered to bring sufficient doses of RES to the lung tissue and the pulmonary route seems the most indicated. Moreover, a local administration presents the advantage of limiting a possible systemic toxicity, therefore improving the safety profile^6^. The present study proposes an effective way to overcome RES low oral bioavailability and therefore to reveal its potential for lung cancer chemoprevention *in vivo*. The efficacy of RES was assessed in the well-established 4-(methylnitrosamino)-1-(3-pyridyl)-1-butanone (NNK)-induced lung carcinogenesis model in A/J mice, mimicking the tobacco-induced lung carcinogenesis in humans^22^. Further *in vitro* investigations provided insights on RES mechanism of action.

## Results and Discussion

### Design of a pharmaceutical formulation for lung delivery and pharmacokinetic study

**Figure 1:**
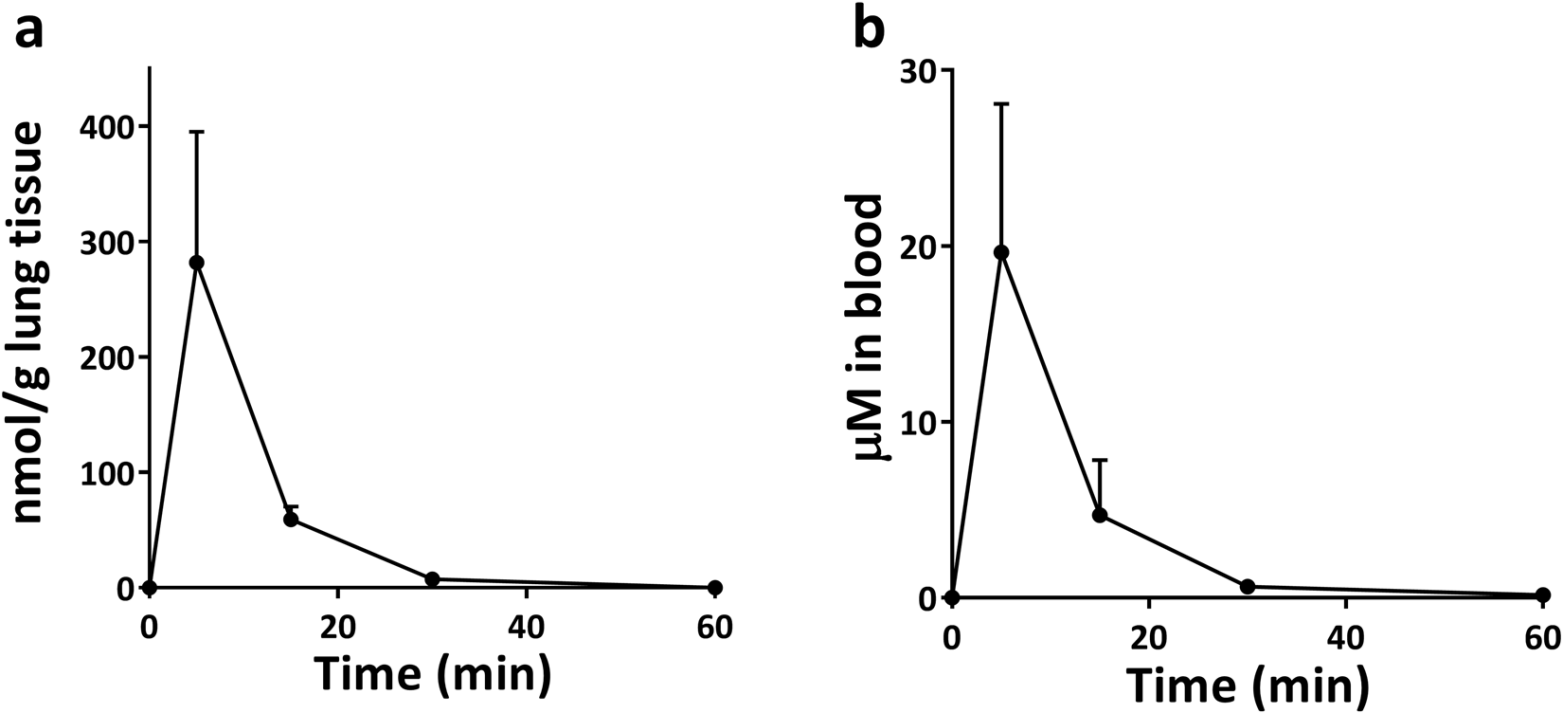
RES content over time in lung tissue (**a**) and in the blood (**b**) after a single intranasal administration of 1.2 mg/mouse RES (n = 3 for each time point).

In an attempt to achieve local administration of RES, a major obstacle resided in its very poor water solubility (0.03 mg/ml). This problem being shared with many other bioactive natural compounds, several strategies have been developed to increase the water solubility of such molecules, going from simple co-solvation with organic solvents (e.g. DMSO) to the use of lipid nanoparticles. Among those strategies, hydroxypropyl-β-cyclodextrin (HPCD), a FDA-approved non-toxic excipient notably used in injectable formulations, led to a huge increase in RES water solubility, reaching up to 38 mg/ml^23^. This excipient was therefore chosen to design the pharmacological formulation for lung administration of RES. A sterile 200 mM hydroxypropyl-β-cyclodextrin (HPCD) saline solution was used to solubilize 24 mg/ml (105 mM) of RES, allowing the administration of a high amount of RES (1.2 mg/mouse) in a limited volume (50 μl) through intranasal instillation. Among the several methods existing to administer a solution to mice lungs, intranasal instillation is probably the easiest and was shown efficient in lung cancer chemoprevention studies^24^. Quantification of RES content in both lung tissues and the blood stream at different time-points showed a peak concentration at 5 min and a substantially decreased concentration at 15 min for both compartments (Fig. 1a,b). Compared to previously published results using the oral route^25^, pulmonary administration was 22.5 times more efficient to bring RES to the lungs, and 2.5 times more efficient to bring RES to the blood stream, reaching a 20 µM blood concentration (Table 1). The shape of the distribution curves displayed however the same quick absorption and clearance of RES from the lungs after pulmonary administration, partly through systemic absorption. Nonetheless, the combination of the formulation with an intranasal mode of administration allowed the exposure of lung tissues to a high amount of RES and could therefore be used for the lung carcinogenesis study.

**Table 1:** Comparison of maximum RES content in the mice lung and blood after oral and pulmonary administrations.

	Oral route^a^	Pulmonary route	Improvement in bioavailability
Administered dose	240 mg/kg	~60 mg/kg	
Lung concentration	50 nmol/g	281 nmol/g	⇨ × 22.5
Blood concentration	32 µM	20 µM	⇨ × 2.5

^a^Data obtained from^25^.

### RES successfully prevented NNK-induced lung carcinogenesis

**Figure 2:**
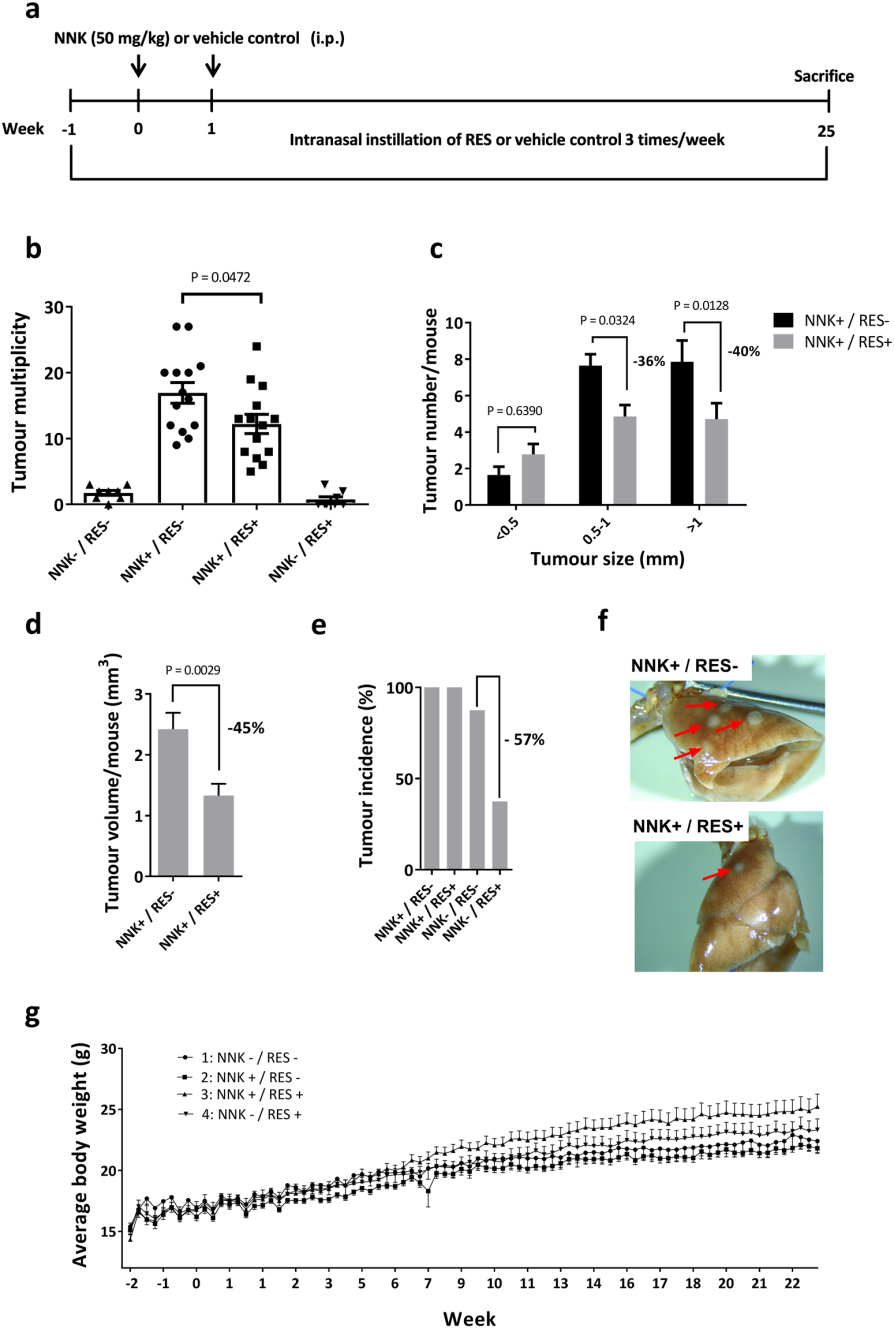
Inhibition of NNK-induced lung carcinogenesis by RES treatment. (**a**) Experimental design of the study. (**b**) Mice were randomized into four groups and received the following treatments: Group 1 (NNK−/RES−); Group 2 (NNK+/RES−); Group 3 (NNK+/RES+); Group 4 (NNK−/RES+). Tumour multiplicity in groups 1–4. Significant reduction was observed in group 3 compared to group 2 (P = 0.0472) (**c**) Tumour size in groups 2 and 3. Significant reduction was observed in tumours with 0.5–1 mm (P = 0.0324) and >1 mm (P = 0.0128) diameter (**d**) Estimated total tumour volume in groups 2 and 3, calculated with the formula *V* = (*D* ∗ *d* ∗ 0.2 ∗ *π*)/6). Significant reduction was observed in group 3 (P = 0.0029) (**e**) Tumour incidence. (**f**) Representative images of lungs from mice of groups 2 and 3. Visible tumours are pointed by red arrows. (**g**) Body weight as a function of time in each group. No obvious sign of toxicity was observed in RES treated groups.

The well-established NNK-induced lung carcinogenesis model in A/J mice was used to evaluate RES effect on lung carcinogenesis (Fig. 2a). Two intraperitoneal injections of NNK resulted in 100% lung cancer incidence after 25 weeks, with up to 27 tumours/mouse. RES treatment significantly reduced both tumour multiplicity (P = 0.0472) and size (P = 0.0324 for tumours of 0.5–1 mm in diameter, P = 0.0128 for tumours bigger than 1 mm) in NNK-induced groups, without however impacting incidence (Fig. 2b–d,f). In non NNK-induced groups, RES treatment decreased spontaneous tumour incidence by 57% (Fig. 2e). No adverse effect was observed in either group as indicated by the body weight curves (Fig. 2g) and the absence of behavioural changes during the study. Therefore, despite its quick clearance, pulmonary administration proved to be an efficient way to administer a high amount of RES to the lungs and revealed its lung cancer chemopreventive properties in a chemically-induced lung carcinogenesis model, in which oral administration has failed.

### RES acted independently of the NF-κB pathway

**Figure 3:**
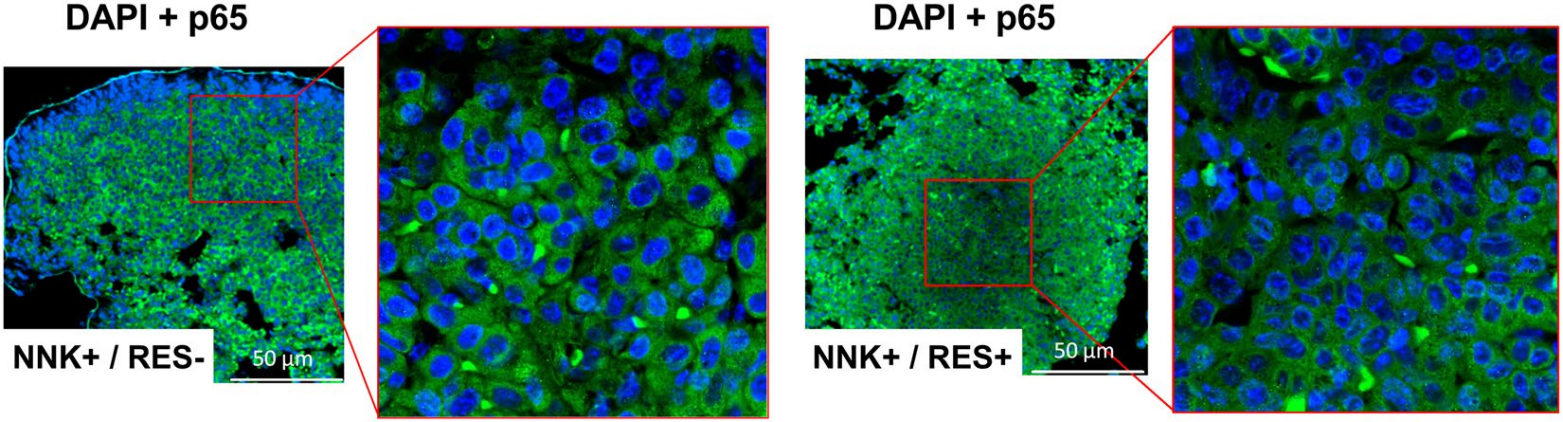
NNK did not induce NF-κB activation *in vivo*. Immunohistochemistry on samples of lung tumours from groups 2 and 3 with NF-κB p65 (stained in green) and DAPI (stained in blue).

To better understand the mechanisms involved in the observed *in vivo* lung cancer chemopreventive activity, RES effect on the NF-κB pathway was first evaluated by looking at the nuclear translocation of p65, a subunit of the transcription factor that translocates upon activation. Surprisingly, immunohistochemical analysis of lung samples from both groups treated with NNK (with and without RES) revealed no p65 nuclear translocation (Fig. 3). A possible explanation is that all observed tumours were adenomas, which are early stage tumours while NF-κB activation was shown in late stage tumours^11^. Therefore, the mechanism of action responsible for the *in vivo* effects of RES is probably independent of its NF-κB inhibitory properties.

### RES increased NNK-induced γ-H2AX phenotype

**Figure 4:**
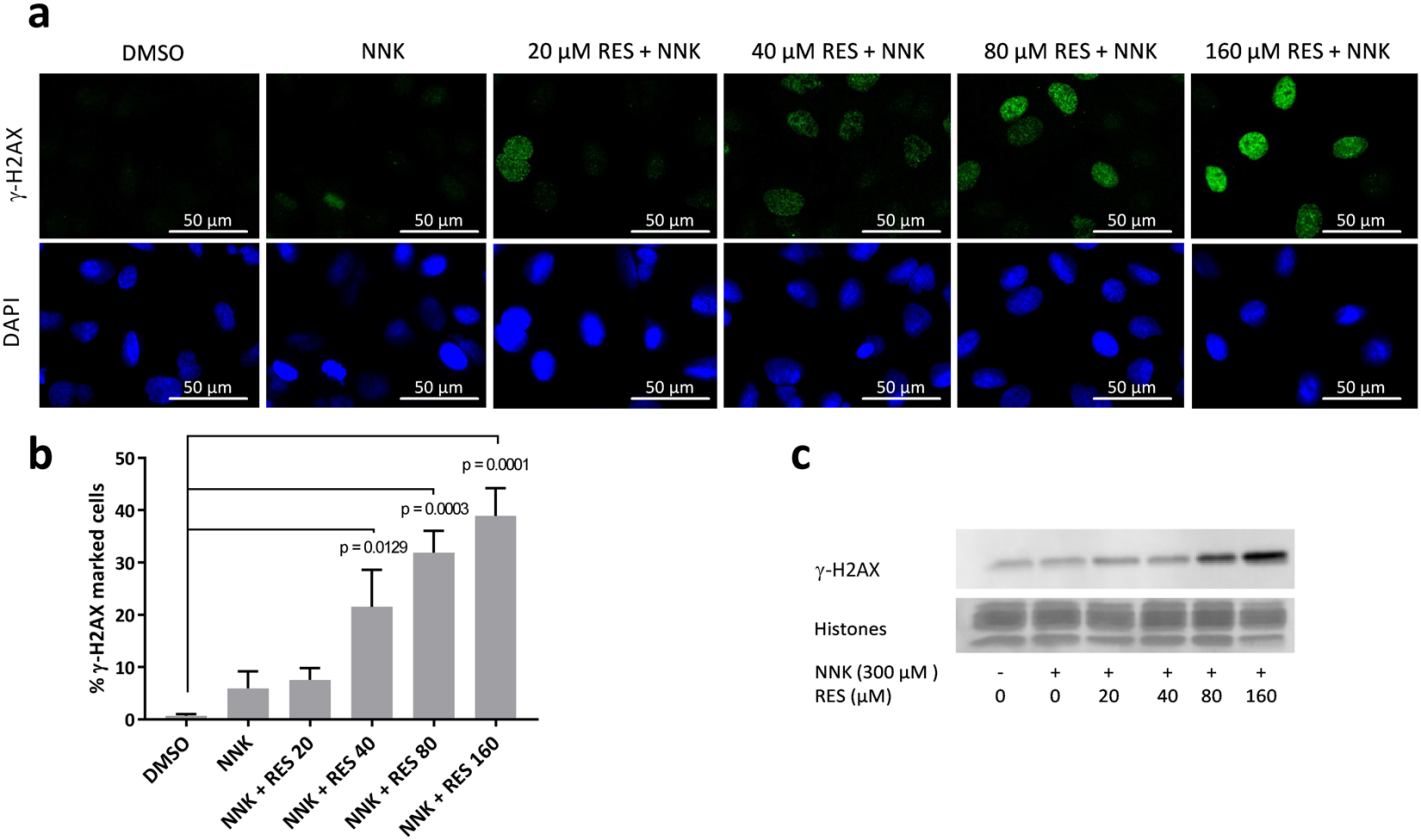
RES induced γ-H2AX protein expression in A549 cells. Immunocytochemistry analyses (**a**) and quantification (**b**) of γ-H2AX marked A549 cells following treatment with DMSO (0.05%), NNK alone, or NNK + RES. Significant γ-H2AX induction was observed after treatment with NNK + RES 40 µM (P = 0.0129), NNK + RES 80 µM (P = 0.0003), and NNK + RES 160 µM (P = 0.0001) compared to DMSO control (n = 3). (**c**) Cropped western blot analyses of γ-H2AX expression in A549 cells following DMSO (0.05%), NNK alone, or NNK + RES treatment. Full lengths blots/gels are presented in Supplementary Fig. S1.

The apparent NF-κB independence of RES mechanism of action led to the investigation of other plausible aspects of NNK-induced carcinogenesis that could have been affected by the treatment. A hypothesis was that RES could act by limiting NNK-induced DNA damage, as such genoprotection was observed *in vitro* against benzo[a]pyrene DNA toxicity^26^. NNK is a procarcinogen that becomes genotoxic after bioactivation by CYP2A6 and CYP2A13, two extrahepatic cytochromes expressed in the lungs^27^. Inhibition of CYP2A6 efficiently protected A/J mice from NNK-induced lung carcinogenesis^28^, highlighting the interest of CYP inhibitors in lung cancer chemoprevention. Interestingly, RES is known to inhibit multiple CYPs including CYP2A6^29^, but its activity on CYP2A13, the most important in NNK bioactivation, is not known. The potential of RES to protect A549 cells from NNK-induced DNA double strand breaks (DSB) was therefore investigated using γ-H2AX as a biomarker. Surprisingly, both western blot and immunocytochemistry results showed that RES promoted NNK-induced γ-H2AX phenotype, suggesting an induction of NNK genotoxicity instead of the expected protection (Fig. 4). Although unexpected, the observed γ-H2AX induction following RES treatment was consistent with recent data from Luo *et al*.^30^. Induction of γ-H2AX being also involved in the apoptosis process^31^, the present observations could be caused by RES proapoptotic activity in A549 cells rather than a real genotoxic effect.

### RES induced apoptosis in A549 cells

**Figure 5:**
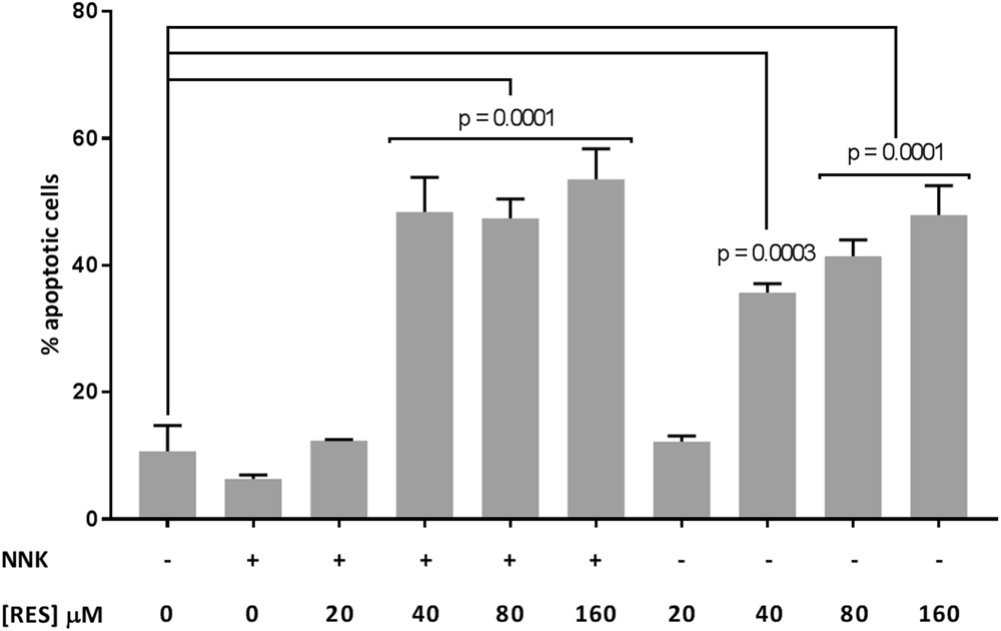
RES induced apoptosis in A549 cells. Apoptosis measurement using Annexin V/PI in A549 cells treated with NNK + RES or RES alone (n = 3). Significant increase in apoptotic cells was observed after treatment with NNK + RES 40 µM (P = 0.0001), NNK + RES 80 µM (P = 0.0001), NNK + RES 160 µM (P = 0.0001), RES 40 µM (P = 0.0003), RES 80 µM (P = 0.0001), and RES 160 µM (P = 0.0001) compared to DMSO control.

Apoptosis induction by RES in A549 cells was evaluated to investigate the link between γ-H2AX expression and apoptosis. Both apoptosis and γ-H2AX induction were observed at the same RES concentrations, suggesting that those two responses to RES treatment were part of a same biological process (Fig. 5). To further understand if RES-induced apoptosis was linked to the NNK-induced DNA damage, apoptosis was measured without NNK treatment. Similar results were obtained with and without NNK treatment, supporting an NNK independent mechanism. These *in vitro* results point at apoptosis induction in lung cancer cells as a potential mechanism of action involved in RES chemopreventive activity. Both γ-H2AX and apoptosis induction occurred at 40 μM, an applicable *in vivo* concentration for the lung compartment, considering that 5 min after administration, the blood concentration was of 20 μM. Because of its involvement in the apoptosis process, the use of γ-H2AX induction as a biomarker of DSB was unfortunately not able to provide clear information about RES effect on NNK-induced genotoxicity. It remains possible that part of the chemopreventive activity of RES was due to CYP2A6 and/or CYP2A13 inhibition, preventing NNK bioactivation. However, the difference in tumour incidence between both groups that did not receive NNK (with and without RES, Fig. 2e), therefore representing spontaneous carcinogenesis, suggests an NNK-independent mechanism, such as the one observed with apoptosis induction. Unfortunately, the design of this study did not allow to observe the proapoptotic effect of RES on lung slices, nor its effect on cell proliferation (analysed using TUNEL and Ki-67 staining, respectively; data not shown). A possible explanation may be that the sacrifice, which happened three days after the last RES treatment, was too late for observing any direct effect of RES.

Overall, these results tend to support the hypothesis that the low oral bioavailability of RES was at least partly responsible for the lack of activity observed in former studies, pointing at other routes of administration as potent ways to overcome it. Even though pulmonary administration may not be the most convenient parenteral route to administer low bioavailability drugs compared to transdermal or sublingual routes, it offers the obvious advantage of reaching the appropriate location. Other parenteral routes should however be investigated in the search for the best activity/convenience ratio that will ensure a satisfactory observance. The transdermal administration could offer the advantage of a continuous drug delivery, allowing long term systemic exposure to the drug instead of the peak shaped pharmacokinetic profile observed with both oral and lung delivery^32^. An improvement of this pharmacokinetic profile through pharmaceutical or chemical engineering to allow a longer exposure to unmetabolized RES could be an interesting way to optimize the *in vivo* activity.

As presented earlier, a major advantage of RES resides in its well-established low toxicity, as it is a food supplement in many countries. This should facilitate the implementation of clinical trials aiming at confirming its effect on lung carcinogenesis. For example, intranasal administration of a RES-carboxymethyl-β-glucan solution was tested in children with allergic rhinitis thrice daily during two month without adverse events^33^. A quick implementation of lung cancer chemoprevention clinical trials seems therefore possible, and should ideally be led on both current and ex-smokers. Indeed, the apparent carcinogen-independent apoptosis induction in cancer cells observed in the present study tends to support RES use in ex-smokers that are no longer exposed to smoke carcinogens, while the described NF-κB and cytochrome inhibitory properties supports its use in current smokers. For ex-smokers, one could easily imagine a nebulizer similar to those used for beta-2-sympathomimetic administration in asthma, as HPCD solutions were shown able to undergo aerosolization with droplet size compatible with lung deposition^34^. While the same device could be used in current smokers, an interesting alternative option could be the administration of RES through electronic cigarette smoking, RES being highly soluble (94 g/l) in propylene glycol^35^, the main constituent of electronic cigarette fluid. A RES containing electronic cigarette could combine the advantage of pharmacological cancer chemopreventive activity with promotion of the transition from conventional tobacco products to electronic cigarettes, potentially safer, although this point remains to be consistently clinically established^36^.

## Methods

### Chemicals and reagents

Hydroxypropyl-β-cyclodextrin was kindly provided by Roquette Frères (Lestrem, France). RES (3,5,4′-trihydroxystilbene) was purchased from APIChem Technology (Hangzhou, China). NNK was purchased from Toronto Research Chemicals (Toronto, Canada). Mouse anti-γ-H2AX (Ser139) antibody and FluorSave reagent were purchased from Merck Millipore (Billerica, MA, USA). Rabbit anti-p65 antibody, Alexa Fluor® 488 conjugated anti-rabbit antibody, HRP-linked anti-rabbit and anti-mouse antibodies were purchased from Cell Signaling Technology (Danvers, MA, USA). Fluorescein isothiocyanate (FITC) conjugated anti-mouse antibody was purchased from Jackson ImmunoResearch (West Grove, PA, USA). Normal goat serum was purchased from Abcam (Cambridge, UK). Histo-clear was purchased from National Diagnostics (Atlanta, GA, USA). Other chemicals were purchased from Sigma-Aldrich (Saint-Louis, MO, USA), and used without further purification.

### Formulation

A 24 mg/ml (105 mM) RES solution was prepared in a sterile 200 mM hydroxypropyl-β-cyclodextrin (HPCD) saline solution (0.9% NaCl) as described elsewhere^23^. In order to facilitate RES inclusion in cyclodextrin, RES powder was added to the HPCD solution, vortexed for 5 min at maximal settings, sonicated for 1 h in an ice bath, and finally vortexed at 1200 rpm for 24 h at room temperature. All experimental steps involving RES were protected from light. The formulation was kept at −20 °C and prepared freshly every week.

### Animals

Animals were treated in accordance with the Swiss federal law on the protection of animals with a protocol approved by the Canton of Geneva (Direction Générale de la Santé, authorization number GE/169/14). A total of 56 female A/J mice of 5–6 weeks old were obtained from Envigo (Huntingdon, UK), randomized upon arrival and kept in the conventional animal facility of the School of pharmaceutical sciences at the University of Geneva.

### Pharmacokinetic study

A total of 12 mice were randomized into 4 different groups. Each isoflurane-anesthetized mouse of each group received 50 μl of a 24 mg/ml RES solution (1.2 mg/mouse, ~60 mg/kg) through intranasal instillation as described elsewhere^37^. Mice from groups 1–4 were sacrificed after 5, 15, 30, or 60 min, respectively. Blood was collected upon sacrifice and lungs were harvested. Samples were snap frozen in liquid nitrogen and kept at −80 °C until sample preparation for UHPLC-UV analysis. To extract RES from the lung tissue, samples were put in liquid nitrogen, grinded in a nitrogen-cooled mortar to obtain a fine powder, and weighed. Then, the powder was mixed in an Eppendorf tube with 1 ml of extraction solution (80% methanol, 17.5% distilled water, 2.5% acetic acid) supplemented with 15 μl of a 15% ascorbic acid solution. The mixture was vortexed for 3 min at maximum settings and centrifuged for 15 min at 17 000 *g* at 4 °C to recover the supernatant. This solid-liquid extraction was repeated three times, and the total supernatant was evaporated under nitrogen flux. The extract was then solubilized in 400 μl of a methanol/water 1:1 solution and analyzed by UHPLC-UV. To extract RES from blood samples, 250 μl of blood were acidified with 7.5 μl acetic acid and mixed with 1.25 ml ethyl acetate. The mixture was vortexed for 2 min at maximal settings and centrifuged for 15 min at 17 000 *g* at 4 °C. The supernatant was recovered and evaporated under nitrogen. The extract was solubilized in 100 μl of methanol and analyzed by UHPLC-UV. RES quantifications were performed using an Acquity UPLC System (Waters, Milford, MA, USA) equipped with an Acquity PDA detector for both lung and blood samples. Samples were injected (3 μl) into a C18 Kinetex column (50 mm × 2.1 mm, 2.6 μm, Phenomenex) and eluted (0.5 ml/min, 30 °C) with a mixture of MeOH and H_2_O both containing 0.1% formic acid. A gradient of 10 to 98% MeOH in 3 min was used, followed by a washing step with 98% MeOH for 1 min 50 s. After the washing step, the column was equilibrated with 10% MeOH during 1 min 50 s before the next injection. PDA absorption spectra were recorded between 210 and 490 nm (1.2 nm steps). RES was identified by comparing its retention time (1.67 min) and UV spectra (maximum absorbance at 306 nm) using a standard. Seven RES solutions of concentrations ranging from 0.1 to 25 µg/ml, prepared in both lung and blood matrixes, were analyzed to build calibration curves. Integration of the peak corresponding to RES provided the area under the curve value that was used for quantification. Linearity of the calibration curves was confirmed by their R^2^ of 0.997 and 0.999, respectively.

### Lung cancer chemoprevention study

NNK was used to induce lung carcinogenesis in A/J mice. A total of 44 mice were randomized into 4 groups, receiving the following treatments: no NNK/no RES (group 1, 8 mice), NNK/no RES (group 2, 14 mice), NNK/RES (group 3, 14 mice), no NNK/RES (group 4, 8 mice). An intraperitoneal injection of either 50 mg/kg of NNK (groups 2 and 3) or saline solution (groups 1 and 4) was administered once on weeks 1 and 2. Mice received either 1.2 mg RES (groups 3 and 4) or vehicle control (groups 1 and 2) through intranasal instillation (50 μl) three times a week starting one week before the first NNK (or saline) injection, and for 26 weeks. At the end of the study, mice were sacrificed, and blood and lungs were harvested for further analysis. Lungs were fixed in 10% formalin, while plasma was separated from whole blood and stored at −80 °C. The lung tumours present at the surface were counted and measured under a dissecting microscope. Tumour volumes were calculated using the formula

$$V=(D\ast d\ast 0.2\ast \pi )/6,$$

where *D* is the longer diameter and *d* is the shorter diameter. Mice were weighted three times a week during the entire study.

### Histology and p65 immunohistochemistry

The lungs of one mouse from groups 2 and 3 were paraffin-embedded and tissue sections were prepared. A section of the lung of each mouse containing several tumours was stained with hematoxylin and eosin for tumour characterization^38^. For p65 immunodetection, a slice of the lung of each mouse was deparaffinized in Histo-clear and antigen retrieved by incubating the slides in 1 mM EDTA pH 8.0 at 98 °C for 20 min. Slides were then cooled on the bench for 30 min, washed twice with PBS for 3 min, and incubated for 5 min with 200 μl of a 3% H_2_O_2_ solution to block endogenous peroxidase. After washing with PBS, the slides were incubated for 1 h at room temperature with the blocking solution (0.1% TBST with 5% normal goat serum), followed by overnight incubation at 4 °C in a humidified chamber with rabbit monoclonal anti-p65 antibody (1:100 in blocking solution). Slides were then rinsed three times for 5 min with TBST, incubated with the fluorescent anti-rabbit antibody (1:100 in blocking solution) for 1 h at room temperature in the dark, counterstained with 0.1 µg/ml DAPI and mounted using 1–2 drops of FluorSave reagent. Fluorescent images were taken on a Nikon A1r spectral confocal microscope (Tokyo, Japan).

### Cell culture

A549 cells (ATCC, Manassas, VA, USA) were cultured in Kaighn’s modification of Ham’s F-12 (F-12K) medium (Thermo Fisher Scientific, Waltham, MA, USA) containing 100 U/mL penicillin, 100 µg/ml streptomycin (Thermo Fisher Scientific) and 10% fetal bovine serum (Biowest, Nuaillé, France), at 37 °C in a 5% CO_2_ atmosphere. Cells were authenticated by ATCC using short tandem repeat analysis in 2007. The cells have been passaged in the user’s laboratory for fewer than 6 months after resuscitation. Absence of mycoplasma contamination was assessed regularly using a MycoAlert mycoplasma detection kit (Lonza, Basel, Switzerland).

### γ-H2AX immunocytochemistry

A549 cells were seeded in clear bottom black 96-well plates (Corning, Corning, NY, USA) (10^4^ cells/well) and incubated overnight at 37 °C in a 5% CO_2_ atmosphere. Cells were then treated with either DMSO, 300 µM NNK, or 300 µM NNK + RES at increasing concentrations during 4 h at 37 °C. After incubation, cells were rinsed with DPBS (Thermo Fisher scientific) and fixed with 4% paraformaldehyde for 20 min. Fixed cells were then permeabilized for 5 min with a 0.1% Triton X-100 solution in DPBS (DPBST), blocked during 30 min with 1% bovine serum albumine in DPBST (blocking solution), and incubated overnight at 4 °C with the mouse monoclonal anti-γ-H2AX antibody (1:400 in blocking solution). Cells were then washed 3 times with DPBST, incubated for 1 h at room temperature in the dark with the FITC conjugated anti-mouse antibody (1:200 in blocking solution), and counterstained with 0.1 µg/ml DAPI for 1 min. Fluorescent pictures were taken on a Nikon A1r spectral confocal microscope and a Cytation 3 imaging multimode plate reader (Biotek, Winooski, VT, USA). Induction of γ-H2AX was quantified in three independent experiments by measuring the fluorescence intensity of the secondary antibody in the nuclear zones defined by the DAPI staining, using the Gen5 3.0 software (Biotek).

### Immunoblot analyses

A549 cells were seeded in 6-well plates (20 × 10^4^ cells/well) and incubated overnight at 37 °C in a 5% CO_2_ atmosphere. Cells were then treated with either DMSO, 300 µM NNK, or 300 µM NNK + RES at increasing concentrations for 4 h at 37 °C, rinsed with 2 ml of PBS containing 2 mM sodium butyrate and scrapped in 500 µl of the same solution. Detached cells were centrifuged at 200 *g* for 10 min at 4 °C, and the supernatant was removed. An acidic extraction was performed to obtain cell lysates concentrated in histones. Cell pellets were mixed in 5-fold their volume of lysis buffer (10 mM HEPES pH 7.9, 1.5 mM MgCl_2_, 10 mM KCl, 0.5 mM DTT and 1.5 mM PMSF) and acidified with HCl (0.2 M final) to selectively solubilize basic proteins such as histones. After 30 min incubation at 4 °C, the lysates were centrifuged at 11 000 *g* for 10 min at 4 °C. The supernatants containing the histones were collected and their protein content was quantified on a Qubit 3.0 fluorometer (Thermo Fisher scientific). Fifteen μg of proteins were loaded on 12.5% SDS-PAGE gels and run for 1.5 h at 100 V. Proteins were then transferred to PVDF membranes (Bio-Rad, Hercules, CA, USA) for 40 min at 20 V in a pH 10, MeOH free transfer buffer (5.82% tris-base, 2.93% glycine). Membranes were blocked for 1 h at room temperature in 5% non-fat milk and incubated overnight at 4 °C with the mouse monoclonal anti-γ-H2AX antibody (1:1000 in 1% non-fat milk). Membranes were then washed three times for 10 min with TBST, incubated for 1 h at room temperature with the HRP-linked anti-mouse antibody (1:1000 in 1% non-fat milk), and revealed through chemiluminescent immunodetection with Pierce ECL western blotting substrate (Thermo Fisher Scientific). The experiment was repeated two times and similar results were obtained.

### Apoptosis assay

Induction of apoptosis was determined using the annexin V-fluorescein isothiocyanate (FITC)/propidium iodide (PI) assay according to the manufacturer’s protocol (Thermo Fisher scientific). A549 cells were seeded in 12-well plates (10^5^ cells/well) and incubated overnight at 37 °C in a 5% CO_2_ atmosphere. Cells were then treated with DMSO, 300 µM NNK, RES at increasing concentrations with or without 300 µM NNK during 48 h at 37 °C. All the cells were harvested, washed with cold PBS, and stained with 5 µl of annexin V-FITC and 1 µl of PI (100 µg/ml) for 15 min in the dark at room temperature. Percentage of apoptotic cells was determined in three independent experiments by counting the number of annexin V-FITC positive cells using an Attune NxT flow cytometer and the dedicated Attune NxT software (Thermo Fisher scientific).

### Statistical analyses

Data are presented as mean ± SEM. Tumour multiplicity in all four groups were compared through a one-way ANOVA, followed by Tukey’s multiple comparison test. Size group comparison for groups 2 and 3 were carried out through two-way ANOVA followed by Sidak’s multiple comparison test. Comparison of total tumour volume/mouse was carried out through unpaired t-test. Percentage of γ-H2AX marked cells as well as apoptotic cells after the different treatments were compared using a one-way ANOVA followed by Dunnett’s multiple comparison test. *P*-values ≤ 0.05 were considered statistically significant. All statistical analyses were performed on GraphPad Prism 6.05.

## Electronic supplementary material

Supplementary information
