# Supplementary material for: Intranasal administration of resveratrol successfully prevents lung cancer in A/J mice
Source: Sci Rep. 2018 Sep 24;8:14257. doi: 10.1038/s41598-018-32423-0 (PMC6155121; doi:10.1038/s41598-018-32423-0)
Supplement: Supplementary file 1 — Supplementary information [file 41598_2018_32423_MOESM1_ESM.pdf]

Intranasal administration of resveratrol successfully prevents lung cancer in A/J mice

Aymeric Monteillier, Aymone Voisin, Pascal Furrer, Eric Allémann, Muriel Cuendet*

School of pharmaceutical sciences, University of Geneva, University of Lausanne, Rue Michel-Servet 1, CH-1211 Geneva 4, Switzerland

* To whom correspondence should be addressed. Tel: +41 22 379 33 86; Email: muriel.cuendet@unige.ch

a

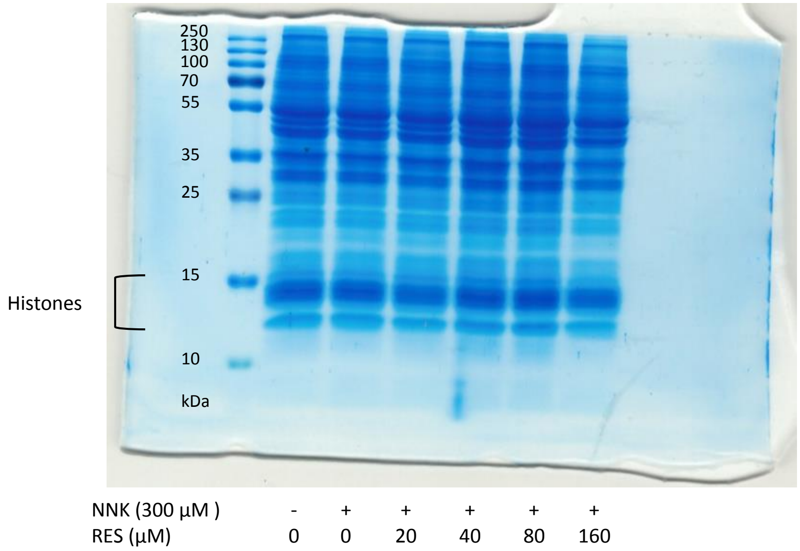

b

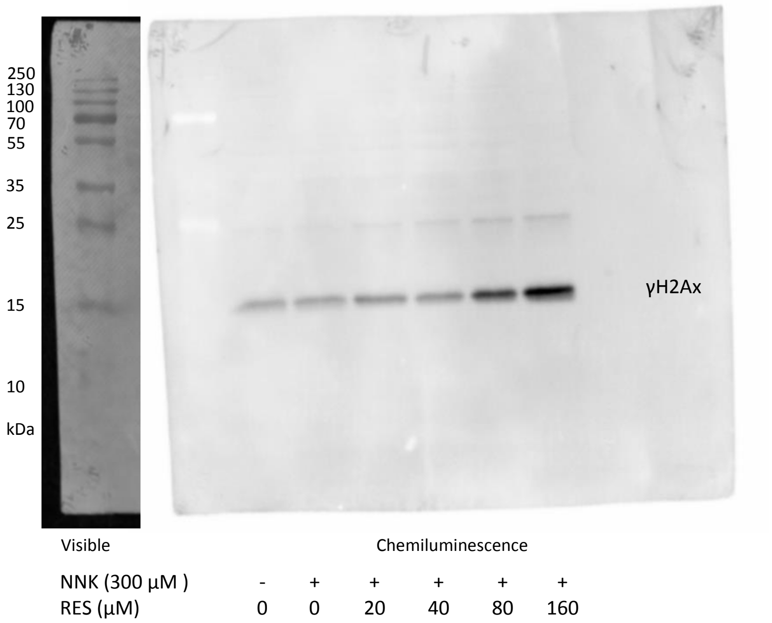

Supplementary Figure S1: Full length Coomassie blue stained gel showing histones bands as a loading control (**a**) and corresponding western blot showing induced γ-H2AX protein expression in A549 cells after RES treatment (**b**).
